# Cucurbitacin E inhibits cellular proliferation and induces apoptosis in melanoma by suppressing HSDL2 expression

**DOI:** 10.1186/s13020-022-00582-y

**Published:** 2022-02-22

**Authors:** Wen-Bei Liu, He-Li Wang, Lei Chen, Biao Tang, Guolin Ke, Shuai Wang, Yin-Qiao Sun, Junting Ma, Da-Lun Lyu

**Affiliations:** 1 Department of Dermato-Venerology and Department of Burn and Plastic Surgery, The First Affiliated Hospital of Wannan Medical College, Jinghu District, Wuhu, 241000 Anhui China; 2 Department of Pharmacology, School of Basic Medical Sciences, Anhui Medical University, Hefei, 230032 Anhui China

**Keywords:** Melanoma, HSDL2, Cucurbitacin E, ERK and AKT pathways, Proliferation and apoptosis

## Abstract

**Background:**

Melanoma is among the most aggressive types of skin malignancy and can have an unpredictable clinical course. Exploration of novel therapeutic targets and their regulators remains essential for the prevention and treatment of melanoma.

**Methods:**

HSDL2 protein levels were examined by immunohistochemistry. The roles of HSDL2 in cell proliferation and apoptosis were identified by CCK-8 and colony formation assays. The function of HSDL2 in cell apoptosis was analysed by flow cytometry. Western blotting, cell proliferation and apoptosis and a xenograft tumour model were utilized to explore the inhibitory functions and mechanisms of CuE in melanoma.

**Results:**

HSDL2 is overexpressed in melanoma and promotes melanoma progression by activating the ERK and AKT pathways. CuE could inhibit the ERK and AKT pathways by decreasing HSDL2 expression; therefore, CuE could inhibit melanoma growth in vitro and in vivo.

**Conclusion:**

HSDL2 may be a promising therapeutic target against melanoma, and CuE can inhibit melanoma by downregulating HSDL2 expression.

**Supplementary Information:**

The online version contains supplementary material available at 10.1186/s13020-022-00582-y.

## Introduction

Skin cancer is the most common type of cancer in dermatology [1], and melanoma is one of the most fatal forms and is the fifth most common cancer in men and the sixth in women [2, 3]. The incidence of melanoma has increased rapidly over past decades [4]. Although advances in surgical resection, radiation, and chemotherapy have improved the survival of cancer patients in recent decades, mortality rates remain high due to tumour recurrence and metastasis [5, 6]. Thus, the exploration of new effective molecular targets on tumour cells and their regulators would be useful for melanoma therapy.

Hydroxysteroid dehydrogenase-like (HSDL) 2 is a member of the short-chain dehydrogenase/reductase (SDR) superfamily that can catalyse the oxidation and reduction of various substrates, such as steroids, sugars, retinoids and fatty acids [7]. Previous studies have shown that HSDL2 is involved in lipid metabolism and the synthesis of cholesterol [8, 9], displaying significant protumour function by promoting cell proliferation and inhibiting apoptosis [10, 11], and has been reported to be associated with some cancers, such as pancreatic cancer, breast cancer, and papillary thyroid carcinoma [12–14]. However, to date, the function of HSDL2 in melanoma remains unclear.

Natural compounds found in vegetables, fruits, and medicinal plants have been considered potential sources of inhibitors for cancer [15]. Cucurbitacin E (CuE) is a member of the cucurbitacin family, which is a group of tetracyclic triterpenoids extracted from cucurbitaceous plants [16]. Accumulated evidence has demonstrated that CuE has effective pharmacological properties, such as lipid reduction, hepatic protection, anti-inflammation, and antitumour activity [17]. The potential anticarcinogenic properties of CuE on diverse tumour types, such as gastric cancer, lung cancer, breast cancer and ovarian cancer, have been well studied [18–21]. However, the potential antitumour function and the underlying mechanisms of CuE in melanoma have not been fully elucidated.

In this paper, we found that HSDL2 promoted cell proliferation and suppressed apoptotic cell death by activating the AKT and ERK signalling pathways and that CuE could slow melanoma growth by inhibiting the AKT and ERK signalling pathways via the downregulation of HSDL2 expression.

## Material and methods

### Reagents and chemicals

HSDL2 (Cat: 15,631-1-AP, 1:1000) and α-tubulin (Cat: 11,224-1-AP, 1:1000) were purchased from Proteintech (Wuhan, China). Bcl-2 (Cat: R22494; 1:1000), cleaved caspase-3 (Cat: 383,726; 1:1000), phospho-ERK (Cat: 301,245; 1:1000) and ERK (Cat: 201,245-4A4, 1:1000) were purchased from ZEN-BIOSCIENCE (Chengdu, China). AKT (Cat: A5031, 1:1000) was obtained from Bimake (Houston, TX, USA). Phospho-AKT (Cat: 4060 T, 1:1000) was purchased from Cell Signaling Technology (Danvers, MA, USA). Cucurbitacin E was obtained from MedChemExpress (Cat: HY-N0417, New Jersey, USA).

### Clinical specimens

Melanoma and adjacent normal tissue samples were obtained from 60 melanoma patients recruited from the First Affiliated Hospital of Wannan Medical College. The melanoma patients were between 31 and 76 years of age (mean, 50.3 ± 18.4 years) at diagnosis. Written informed consent was obtained from each patient, and the experimental protocol was approved by the Ethics Committee of the First Affiliated Hospital of Wannan Medical College. The clinicopathological characteristics of the samples are summarized in Table 1.

**Table 1:** Summary of clinicopathological characteristics

Characteristics	Category	Frequency
Gender	Men/Women	28/32
Age group	≤ 50/ > 50	26/34
Clinical stage	I + II/ III + IV	40/20
Tumor thickness	≤ 1/ > 1	37/23

### Cell culture and lentivirus infection

A375 human melanoma cell lines were purchased from the Cell Bank of the Chinese Academy of Sciences (Shanghai, China) and cultured in Dulbecco′s modified Eagle′s medium containing 10% foetal bovine serum and 100 U/ml penicillin/streptomycin (all from Sangon Biotech, Shanghai, China) at 37 °C in a 5% CO_2_ incubator.

HSDL2-overexpressing lentivirus and shRNA targeting HSDL2 lentivirus was obtained from General Biol (Anhui, China). The sequences were as follows: human HSDL2, shRNA: 5′-CCA GAA GCA GTT AGC AAG AAA-3′; scrambled shRNA, shCtrl: 5′-TTC TCC GAA CGT GTC ACG T-3′. Briefly, cells were seeded in 6-well plates and cultured at 37 °C and 5% CO2 for 24 h. The lentiviral particle suspension was added to the plate (multiplicity of infection = 5, 5 µl lentivirus per well). Following infection at 37 °C for 12 h, the culture medium was replaced. The cells were cultured for 72 h, and infection efficiency was determined by real-time reverse transcription quantitative polymerase chain reaction (RT–qPCR) and western blotting assays.

### RT–qPCR

Total RNA was extracted with TRIzol reagent (Beyotime, R0016) according to the manufacturer′s instructions. Reverse transcription was performed using Moloney murine leukaemia virus reverse transcriptase (Promega, Madison, Wisconsin, USA) according to the manufacturer′s protocol along with oligo(dT) primers (Sangon Biotech) to obtain cDNA. HSDL2 mRNA expression was examined with qPCR. The reaction consisted of 1 × SYBR Green Master Mix (Sangon Biotech) and 0.06 µM each primer under the following conditions: 94 °C for 5 min, followed by 40 cycles of 94 °C for 15 s and 58 °C for 40 s. Each sample was run in triplicate. The forward and reverse primers were synthesized by Sangon Biotech and had the following sequences: HSDL2, 5′-AAG CCA CTC AAG CAA TCT ATC TG-3′ and 5′-GCT CTC CAT ATC CGA CAT TCC C-3′; and glyceraldehyde 3-phosphate dehydrogenase (GAPDH), 5′-TGA CTT CAA CAG CGA CAC CCA-3′ and 5′-CAC CCT GTT GCT GTA GCC AAA-3′. The relative HSDL2 expression level was normalized to that of GAPDH and quantitated with the 2^−ΔΔCT^ method.

### Western blot analysis

Cells were washed with phosphate-buffered saline (PBS) and then lysed in lysis buffer composed of 100 mM Tris–HCl (pH 6.8), 0.15 M NaCl, 5 mM EDTA (pH 8.0), 1% Triton X-100, 5 mM dithiothreitol, and 0.1 mM phenylmethylsulfonyl fluoride for total protein extraction. Protein concentration was determined with the BCA Protein Assay Kit (Sangon Biotech); 20 μg of the cell solution was mixed with 5 × loading buffer and separated by sodium dodecyl sulfate–polyacrylamide gel electrophoresis on a 12.5% acrylamide gel. The proteins were transferred to a polyvinylidene difluoride membrane (Amersham Biosciences, Little Chalfont, UK) that was blocked with 5% skim milk dissolved in Tris-buffered saline with 0.1% Tween-20 (TBST) for 1 h at room temperature and then incubated with primary antibodies overnight at 4 °C. After washing three times with TBST, the membrane was incubated with horseradish peroxidase (HRP)-conjugated secondary antibody (Santa Cruz Biotechnology), and immunoreactivity was detected using an ECL Western Blotting Substrate kit (Pierce, Rockford, IL, USA).

### Colony formation assay

A375 cells at a density of 1000 cells/well were resuspended and seeded into six-well plates. After culturing for two weeks, the colonies were fixed with 4% paraformaldehyde for 15 min at room temperature and stained with 0.2% crystal violet for 20 min (Solarbio, Beijing) at room temperature. The number of colonies visible to the naked eye (each colony contained > 50 cells; 1 × magnification) was counted using ImageJ v2 software (ImageJ; National Institutes of Health).

### CCK-8 assay

Cell proliferation was also analysed with the Cell Counting Kit (CCK)-8 assay (Solarbio, Beijing). A375 cells were collected, washed, resuspended, counted using a haemocytometer, added to 96-well plates at 2000 cells/well and cultured at 37 °C and 5% CO_2_. Cell proliferation was analysed every day for 4 days. Briefly, 10 μg CCK-8 solution (5 mg/ml) was added to the cells (10 μl/well), followed by incubation for 2 h. Finally, absorbance at 450 nm was measured with a microplate reader (DeTie, Nanjing, China).

### Cell apoptosis analysis using flow cytometry

Apoptotic cells were detected using an Annexin V-FITC Apoptosis Detection Kit (eBioscience, San Diego, CA, USA) according to the manufacturer′s instructions. Cells were harvested and washed with PBS. Cell suspensions were prepared at a final density of 1 × 10^6^/ml, and a 100-μl aliquot was mixed with 5 μl annexin V-FITC and 5 μl PI staining solution, followed by incubation at room temperature for 10–15 min. Apoptotic cells were detected by flow cytometry (Beckman Coulter, Inc.), and the apoptosis data were analysed using FlowJo v10 software (FlowJo LLC).

### Immunohistochemistry

Tissue samples were fixed in 4% paraformaldehyde, embedded in paraffin, and sectioned at a thickness of 5 μm. Immunohistochemistry staining was analysed by Wuhan Servicebio Technology Co., Ltd. The sections were examined and photographed under a microscope (magnification, 200×; Olympus Corporation) in five random fields of view per sample, and an experienced pathologist test the sections. The expression level of HSDL2 was divided into 4 groups based on the staining intensity (0, negative; 1, low; 2, medium; and 3, high). In addition, the ratio of stained cells was as follows: 0, 0% stained cells; 1,1–25% stained cells; 2, 26–50% stained cells; and 3, 51–100% stained cells. A staining index (score, 0–12) was examined by the staining intensity × the score for the positive area.

### Tumour growth in vivo assay

Female BALB/c nu/nu athymic nude mice (7–9 weeks of age) were purchased from Beijing Vital River Laboratory Animal Technology Co., Ltd. The housing environment was kept between 16 and 26 °C with relative humidity between 30 and 70% under a regular 12-h light, 12-h dark cycle. All experimental procedures were approved by the Institutional Animal Care and Use Committee (IACUC) of the First Affiliated Hospital of Wannan Medical College.

For the tumour growth assay, 12 female BALB/c nu/nu athymic nude mice were randomly divided into two groups (n = 6/group), and then 1 × 10^7^ A375 cells stably transfected with control or HSDL2 shRNA lentivirus were subcutaneously injected into the right flanks of female nude mice. After 4 weeks, the experiments were finished, and the mice were sacrificed and dissected at the endpoint. Then, the volume and weight of the tumours were measured.

For CuE treatment in vivo, 1 × 10^7^ A375 cells were subcutaneously injected into the right flanks of female nude mice. When the tumour size reached approximately 0.5 cm in diameter, the mice were assigned into two groups (n = 6/group) by a random numbered table: (1) Control group injected intraperitoneally with 100 μl PBS; (2) CuE group (intraperitoneally administered with CuE, 5 mg/kg) one times per four days. After 3 weeks, the mice were sacrificed and dissected at the endpoint, and then, the volume and weight of the tumours were measured. In addition, the nude mice were divided into four group (n = 6/group): (1) Control group; (2) CuE group; (3) HSDL2 group; (4) HSDL2 + CuE group. 1 × 10^7^ A375 cells stably transfected with control or HSDL2-overexpressing lentivirus were subcutaneously injected into the right flanks of female nude mice, and then the tumour-bearing mice were intraperitoneally injected with CuE (5 mg/kg) one times per four days. After 4 weeks, the mice were sacrificed and dissected at the endpoint, and then, the volume and weight of the tumors were measured.

### Statistical analysis

All experiments were performed with at least three replicates. SPSS v21.0 software (SPSS Inc., Chicago, IL, USA) was used for statistical analysis. Data are presented as the mean ± standard deviation of three independent experiments. One-way analysis of variance or unpaired two-tailed Student′s t test was used for data. P < 0.05 was considered statistically significant.

## Result

### HSDL2 expression in melanoma and adjacent normal tissues

**Fig. 1:**
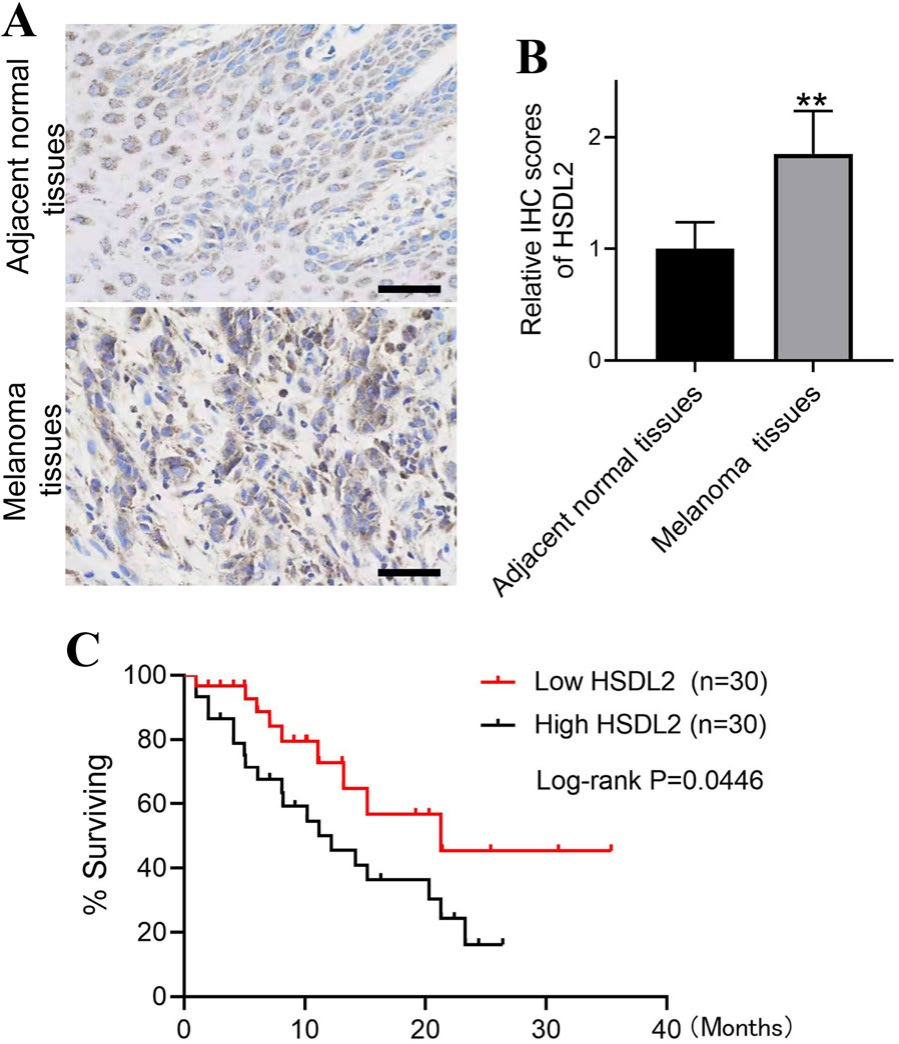
HSDL2 protein expression in melanoma and adjacent normal tissue samples detected by immunohistochemistry. **A** Representative image of HSDL2 IHC staining in the 60 paired melanoma tissue and adjacent normal tissues (scale bar = 200 μm). **B** The expression of HSDL2 in melanoma tissue and adjacent normal tissues based on the results of IHC was quantified. **C** Kaplan–Meier survival curve of HSDL2 in melanoma according to the expression of HSDL2. The results are presented by the mean ± SEM; **P < 0.01, compared with the control group

HSDL2 expression was examined in melanoma and adjacent normal tissues by immunohistochemistry. HSDL2 was upregulated in melanoma (Fig. 1A), which was confirmed by the higher rate of immunopositivity relative to nontumour tissue (Fig. 1B). Importantly, Kaplan–Meier survival curve analysis demonstrated that there was a marked association between higher expression of HSDL2 and worse melanoma patient survival (Fig. 1C).

### Function of HSDL2 in melanoma

**Fig. 2:**
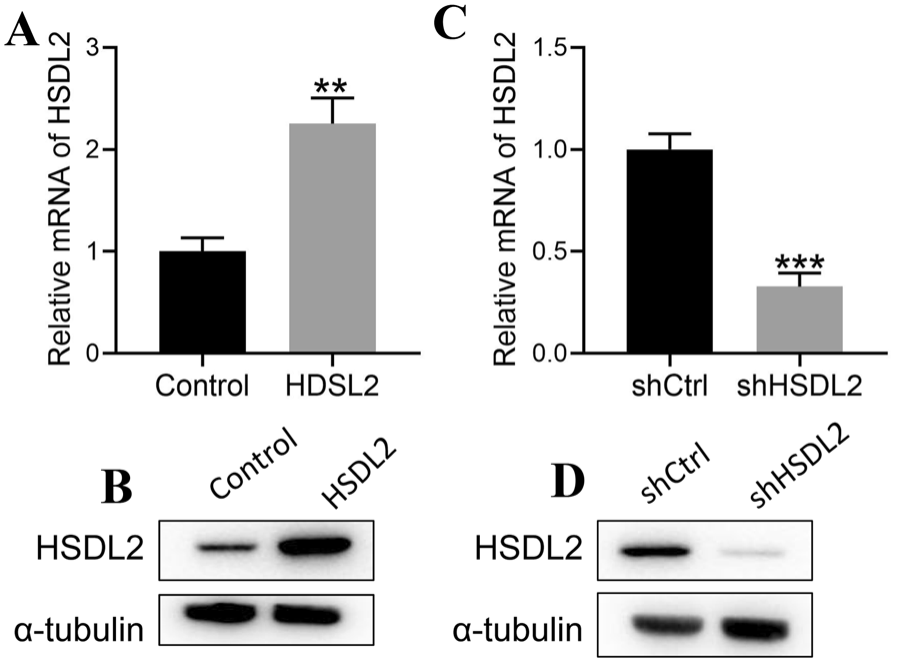
Efficiency of HSDL2 expression in A375 cells using HSDL2-overexpressing lentivirus or shRNA-targeting HSDL2 lentivirus. **A**, **B** The expression of HSDL2 was examined in HSDL2-overexpressing A375 cells via RT–qPCR and western blotting. **C**, **D** The expression of HSDL2 was detected in HSDL2-silenced A375 cells via RT–qPCR and western blotting. The experiments were repeated three times. The results are presented as the mean ± SEM; **P < 0.01 and ***P < 0.001, compared with the control group

To investigate HSDL2 function in melanoma, A375 cells were infected with an HSDL2-overexpressing lentivirus or shRNA targeting HSDL2 lentivirus. The expression of HSDL2 in A375 cells after infection is shown in Fig. 2. HSDL2 lentivirus infection elevated HSDL2 expression (Fig. 2A, B), while shRNA-HSDL2 infection inhibited HSDL2 expression (Fig. 2C, D). The above results demonstrated that HSDL2 could be successfully overexpressed or knocked down in A375 cells.

**Fig. 3:**
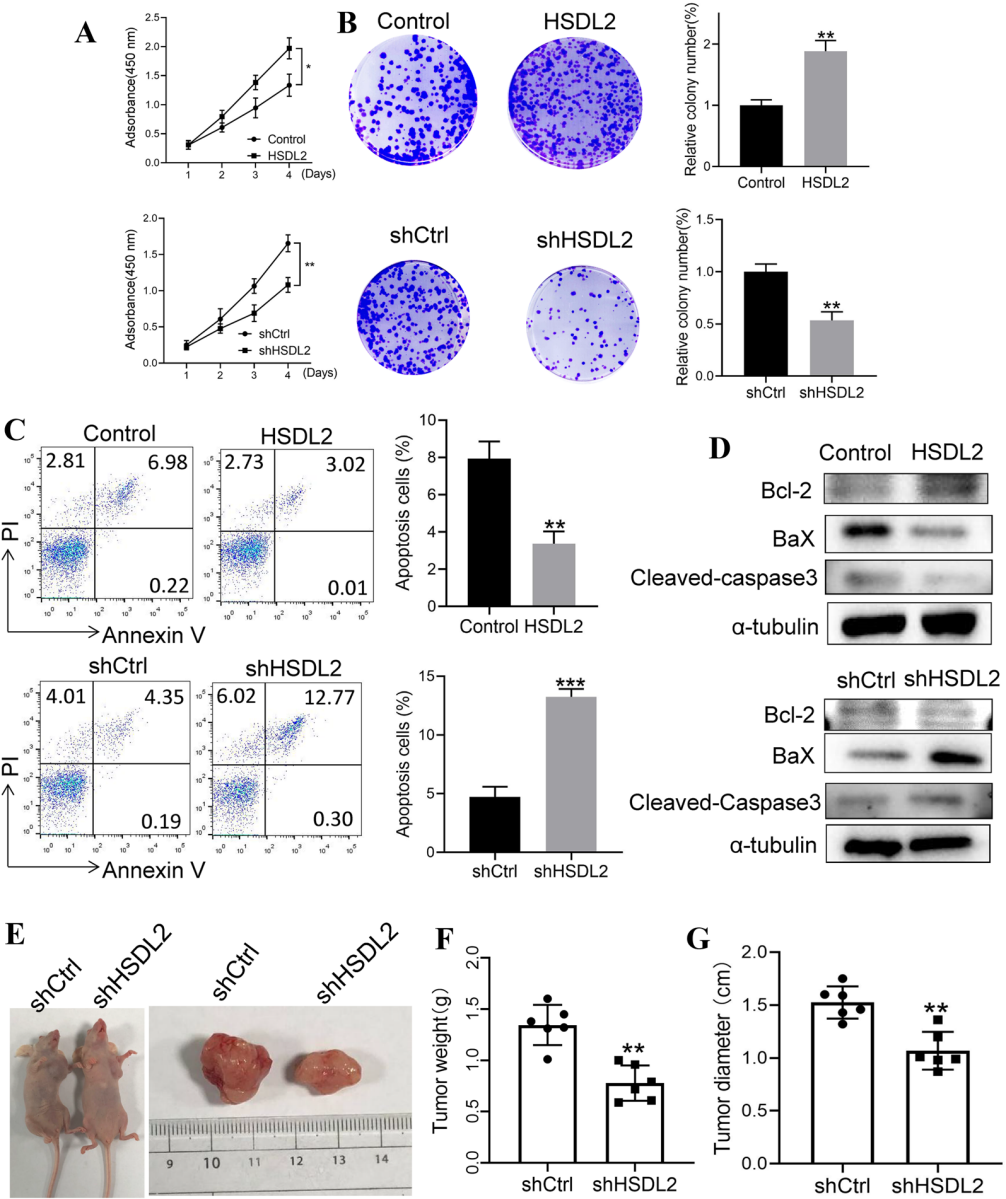
HSDL2 promotes the growth of melanoma in vitro and in vivo. HSDL2 was overexpressed or silenced in A375 cells, and **A** CCK-8 assays were performed. **B** Colony forming assays were performed. Imaging and quantification of colony formation assays were performed between HSDL2-silenced A375 cells or HSDL2-overexpressing A375 cells and control cells. **C** Apoptotic cells were tested by FACS analysis using an Annexin V-FITC Apoptosis Detection Kit. **D** Apoptosis markers (BaX, Bcl-2 and cleaved-Caspase3) were detected by western blotting. **E**–**G** A375 cells with silenced HSDL2 were subcutaneously injected into nude mice. After 4 weeks, the mice were sacrificed and dissected at the endpoint. Tumour growth and weight were examined (n = 6/group). The experiments were repeated three times. All data represent the mean ± SEM, *P < 0.05, **P < 0.01 and ***P < 0.001, compared with the control group

Next, CCK-8 assays and colony formation assays were used to evaluate cell proliferation. Upregulation of HSDL2 promoted A375 cell proliferation, while downregulation of HSDL2 inhibited cell proliferation (Fig. 3A, B). Subsequently, apoptosis assays were also used to test whether HSDL2 could affect apoptosis in addition to inhibiting proliferation. The apoptotic fraction was decreased in HSDL2-overexpressing cells relative to control cells, while the apoptotic fraction was increased in HSDL2-depleted cells relative to control cells (Fig. 3C). Additionally, the relative apoptotic proteins were also investigated, and the results shown in Fig. 3D, overexpression of HSDL2 in A375 cells significantly enhanced the expression of Bcl2, markedly inhibited the expression of BaX and reduced the levels of cleaved caspase-3 relative to control cells, while the expression of Bcl2 was also downregulated, and the levels of BaX and cleaved caspase-3 were increased in HSDL2-depleted cells.

Finally, a subcutaneous tumour xenografting assay in BALB/c nude mice demonstrated that stably silencing HSDL2 in A375 cells significantly suppressed tumour growth. Much smaller tumours were formed by HSDL2-depleted A375 cells, and the average tumour weight and volume at the end of the experiment were markedly decreased in HSDL2-depleted cells relative to control cells (Fig. 3E–G). Taken together, these in vitro and in vivo results suggested that HSDL2 promotes melanoma cell proliferation and inhibits melanoma cell apoptosis.

### HSDL2 knockdown inhibited the AKT and ERK signaling pathways in melanoma cells

**Fig. 4:**
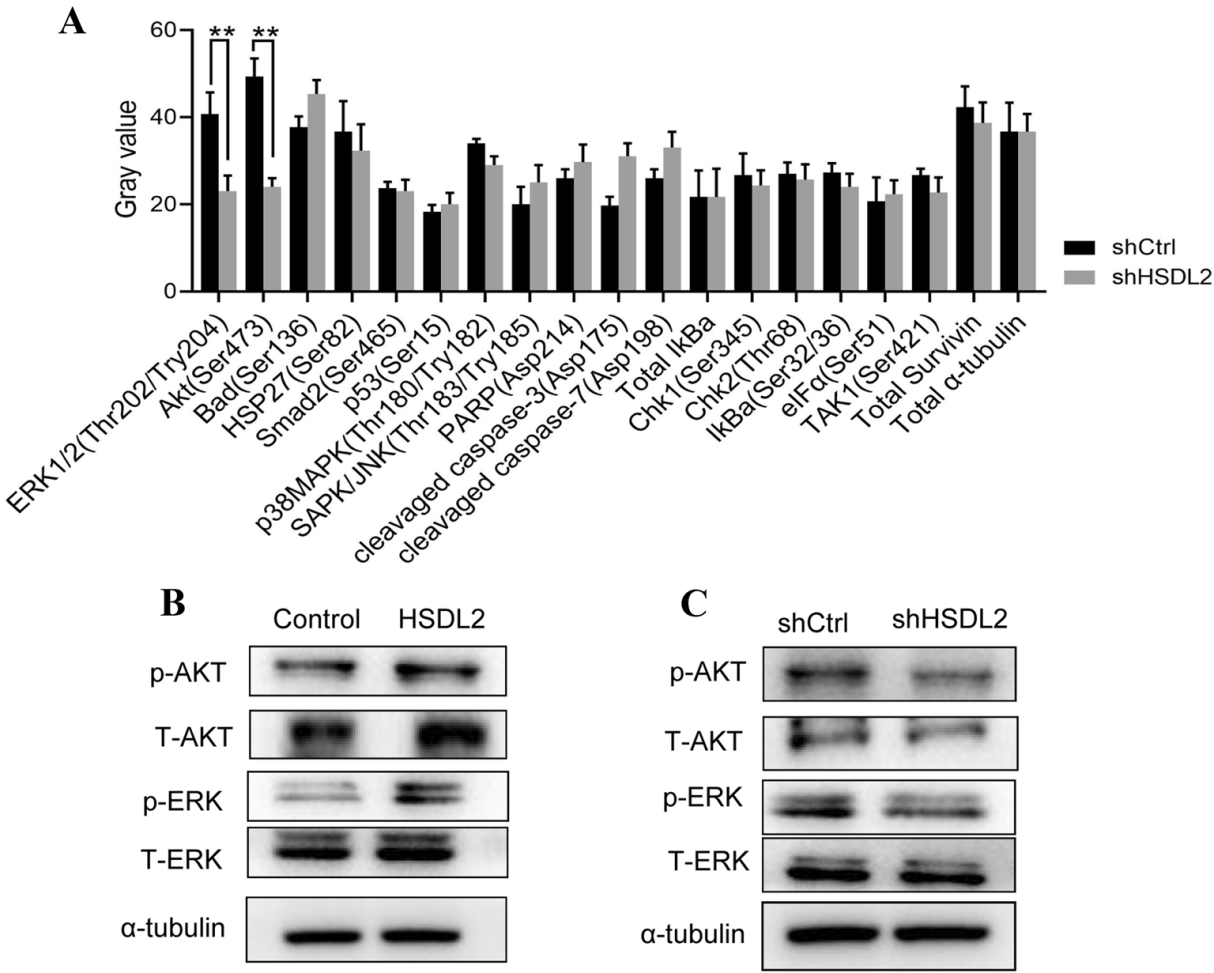
HSDL2 promotes melanoma through the ERK and AKT signalling pathways. A375 cells were infected with shCtrl or shHSDL2 lentivirus. **A** Phosphorylation of 17 signalling proteins was analysed with the PathScan Stress and Apoptosis Signaling Antibody Array, and the image pixel intensity ratio of signalling molecules is shown. **B** HSDL2 was overexpressed in A375 cells, and the ERK and AKT signalling pathways were determined by western blotting. **C** HSDL2 was silenced in A375 cells, and the ERK and AKT signalling pathways were examined by western blotting. Data represent one of three independent experiments. **P < 0.01, compared with the control group

To investigate the mechanisms by which HSDL2 regulated melanoma growth, we examined the activation of multiple signal transduction pathways involved in cell growth and survival in A375 cells after HSDL2 knockdown using the PathScan Stress and Apoptosis Signaling Antibody Array Kit and RTK Signaling Antibody Array Kit. HSDL2 silencing markedly reduced the phosphorylation of ERK1/2 (Thr202/Tyr204) and AKT (Ser473) relative to cells infected with shCtrl lentivirus (Fig. 4A), suggesting that cell growth inhibition in the absence of HSDL2 is due to inactivation of ERK and Akt signalling. The phosphorylation levels of ERK1/2 and AKT were also verified in HSDL2-overexpressing or HSDL2-depleted cells. As shown in Fig. 4B, C, the upregulation of HSDL2 significantly enhanced the phosphorylation levels of ERK1/2 and AKT relative to control cells, while the downregulation of HSDL2 markedly suppressed the phosphorylation levels of ERK1/2 and AKT relative to the control cells. These results showed that HSDL2 promotes proliferation and inhibits apoptosis in melanoma by activating the ERK1/2 and AKT pathways.

### HSDL2 is a target of cucurbitacin E

**Fig. 5:**
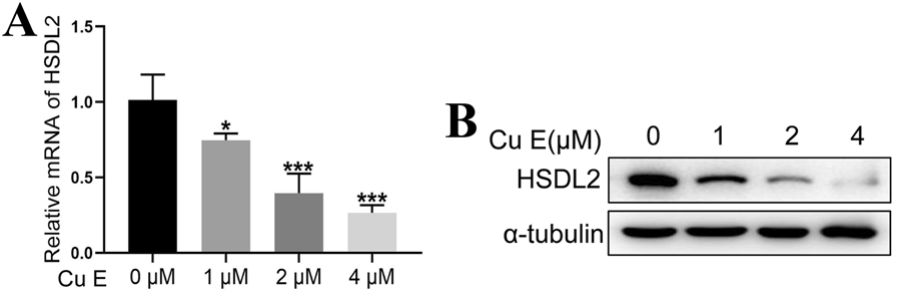
CuE inhibits HSDL2 expression. A375 cells were treated with different concentrations of CuE, and the mRNA and protein levels were examined by RT-qPCR (**A**) and western blotting (**B**). The experiments were repeated three times, and the results of other two repeated experiments on western blotting about HSDL2 expression were showed in Additional file 1: Fig. S1. All data represent the mean ± SEM, *P < 0.05, **P < 0.01 and ***P < 0.001, compared with the 0 μM CuE treatment group

Since HSDL2 had a positive effect on melanoma growth, we attempted to identify a regulator of HSDL2 expression. Hence, we screened some compounds from a natural compound library to assess whether they could regulate the expression of HSDL2. As depicted in Fig. 5A, B and Additional file 1: Fig. S1A, B, the results from RT-qPCR and western blotting assays demonstrated that CuE could inhibit the mRNA and protein expression of HSDL2.

### Cucurbitacin E impedes proliferation and induces apoptosis in melanoma cells

**Fig. 6:**
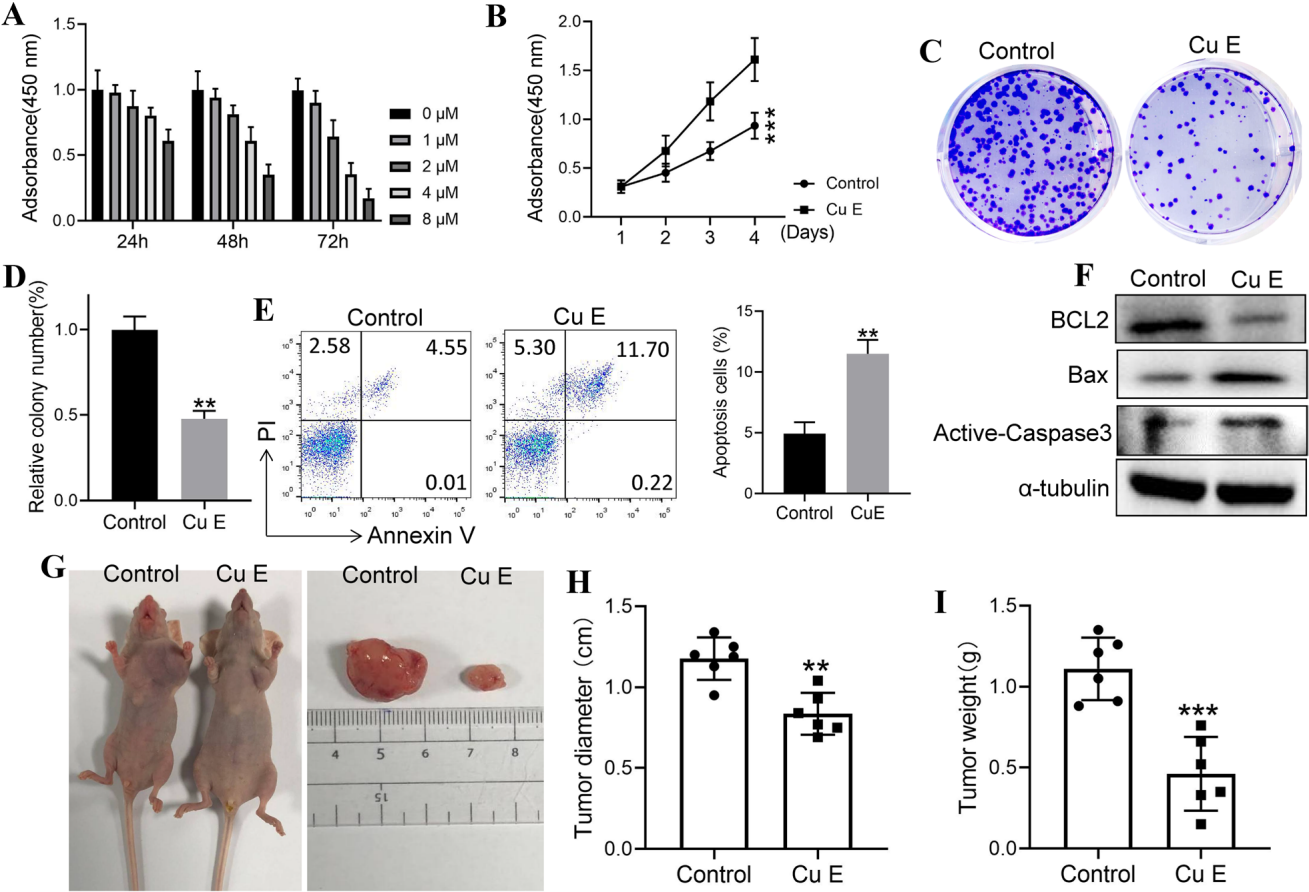
CuE inhibits the growth of melanoma in vitro and in vivo. The inhibitory function of CuE in the proliferation of A375 cells was examined in vitro (**A**), and then 4.0 μM CuE was used to analyse the proliferation rate by CCK-8 assay (**B**). 2.0 μM CuE was used to analyse the colony formation ability by colony forming assay (**C**, **D**). A375 cells were treated with CuE (2.0 µM), and 24 h after treatment, cells were analysed by FACS for cell apoptosis (**E**). Immunoblotting analysis of Bcl2, BaX and cleaved caspase-3 in A375 cells treated with CuE (**F**). A375 cells were subcutaneously injected into nude mice. When the tumour size reached approximately 0.5 cm in diameter, the tumour-bearing mice were intraperitoneally injected with CuE (5 mg/kg). For 3 weeks, the mice were sacrificed and dissected at the endpoint. Tumour growth and weight were examined (n = 6/group, **G**–**I**). The experiments were repeated three times. All data represent the mean ± SEM; **P < 0.01 and ***P < 0.001 compared with the control group

Cell proliferation and apoptosis assays were performed to confirm the anticancer effects of cucurbitacin E. A CCK-8 assay was performed with A375 cells exposed to different doses of CuE (0, 1, 2, 4 and 8 μM). As shown in Fig. 6A, B, CuE inhibited A375 cell proliferation in a dose- and time-dependent manner, and the marked death of A375 cells was induced by 4 μM CuE. Therefore, CuE at a dose of 2 μM was used for subsequent experiments. Colony formation assays showed that CuE remarkably reduced the number of colonies formed by A375 cells (Fig. 6C, D). Next, the effect of CuE on apoptosis in A375 cells was tested by flow cytometry and relative apoptotic protein assays. The results showed that after treatment with CuE, the number of apoptotic cells had significantly increased (Fig. 6E). Similarly, the expression of Bcl2 was also downregulated, and the levels of BaX and cleaved caspase-3 were upregulated in the cells treated with CuE at 48 h (Fig. 6F). In addition, the antitumour activity of CuE in vivo was also assessed using xenografts in BALB/c nude mice, as shown in Fig. 6G–I. CuE significantly slowed tumour growth, and the average weight and volume of tumours at the end of the experiment in the CuE group were less than those in the control group. Taken together, these data suggested that CuE treatment significantly inhibits melanoma growth by suppressing melanoma cell proliferation and enhancing melanoma cell apoptosis.

### Cucurbitacin E suppresses proliferation and induces apoptosis in melanoma cells by downregulating HSDL2

**Fig. 7:**
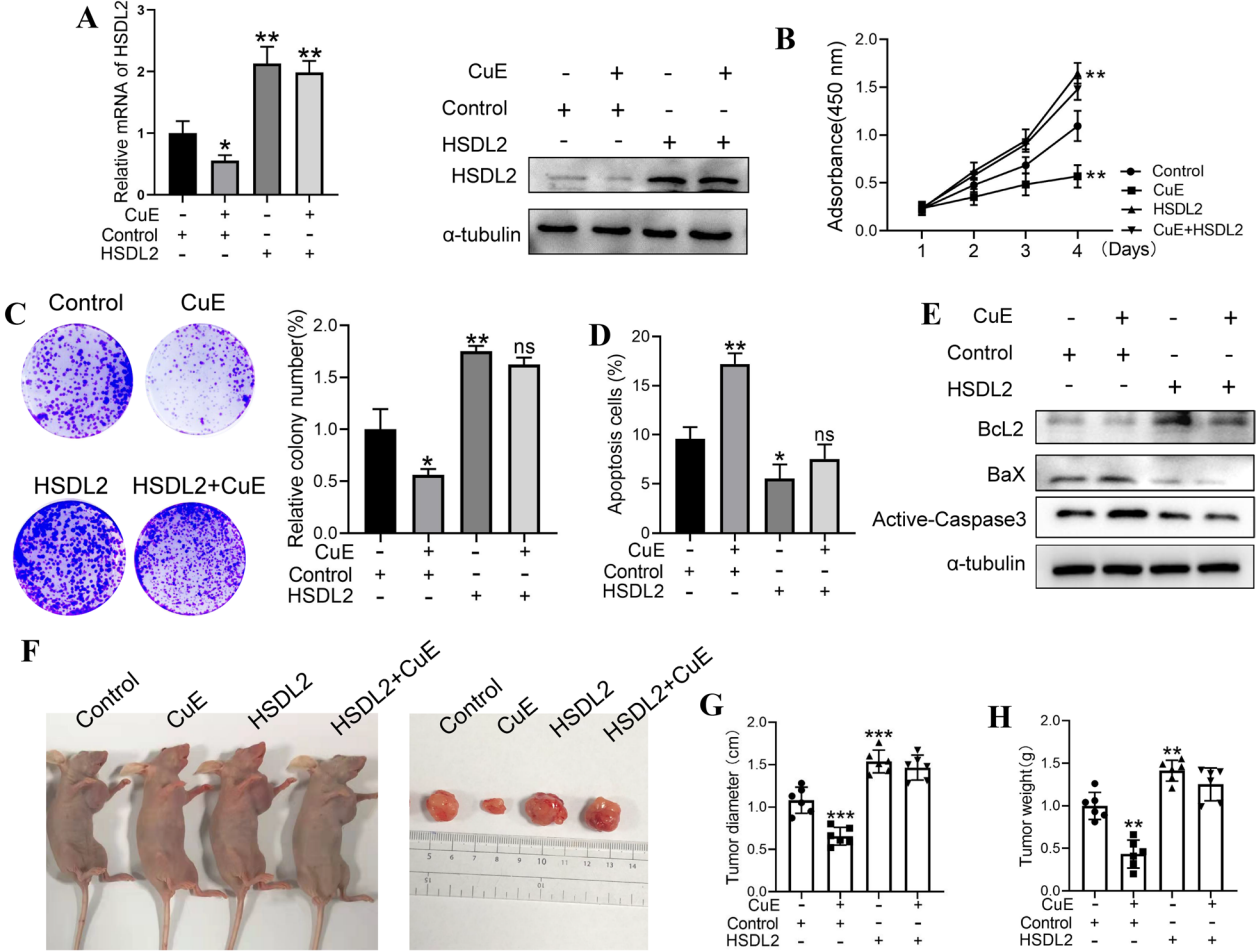
CuE slows melanoma cell growth by mediating HSDL2. A375 cells with or without HSDL2 overexpression were treated with CuE, and the mRNA and protein levels of HSDL2 were tested by RT–qPCR and western blotting (**A**). The proliferation-inhibiting ability of CuE in HSDL2-overexpressing A375 cells was analysed by CCK-8 assay (**B**) and colony forming assay (**C**). The apoptosis of A375 cells with or without HSDL2 overexpression treated with CuE was analysed by flow cytometry (**D**). The levels of Bcl2, BaX and cleaved caspase-3 in A375 cells with or without HSDL2 overexpression treated with CuE were analysed by western blotting (**E**). A375 cells with or without HSDL2 overexpression were subcutaneously injected into nude mice, and then the tumour-bearing mice were intraperitoneally injected with CuE. After 4 weeks, the mice were sacrificed and dissected at the endpoint. Tumour growth and weight were examined (n = 6/group, **F**–**H**). Experiments were repeated three times. All data represent the mean ± SEM, *P < 0.05, **P < 0.01 and ***P < 0.001, compared with the control group

Our results showed that CuE effectively suppressed the growth of melanoma cells and inhibited the expression of HSDL2. However, whether CuE displays its antitumour activity through mediating the level of HSDL2 remained unknown. Thus, we treated HSDL2-overexpressing A375 cells with CuE. RT–qPCR and western blot assays showed higher HSDL2 expression in HSDL2-overexpressing cells treated with CuE than in cells treated with CuE alone (Fig. 7A). The data concluded from CCK-8 assays that HSDL2-overexpressing cells had heightened cell abilities compared to cells treated with CuE alone after culture with CuE for 48 h (Fig. 7B). Subsequently, a colony formation assay demonstrated that the inhibitory effect of CuE on clone formation was also weakened after HSDL2 upregulation (Fig. 7C). In addition, the number of apoptotic cells was also decreased in HSDL2-overexpressing cells treated with CuE (Fig. 7D). Similarly, the expression of Bcl2 was also upregulated, and the levels of BaX and cleaved caspase-3 were suppressed in the HSDL2-overexpressing cells treated with CuE (Fig. 7E). Moreover, in the subcutaneous tumour xenografting assay, upregulation of HSDL2 promoted tumour growth, and the overexpression of HSDL2 slowed the antitumour activity of CuE (Fig. 7F–H). There were no changes in the average weight or volume of tumours between HSDL2-overexpressing cells treated with CuE and cells treated with CuE alone (Fig. 7F–H).

### CuE inhibits the AKT and ERK signalling pathways in melanoma cells

**Fig. 8:**
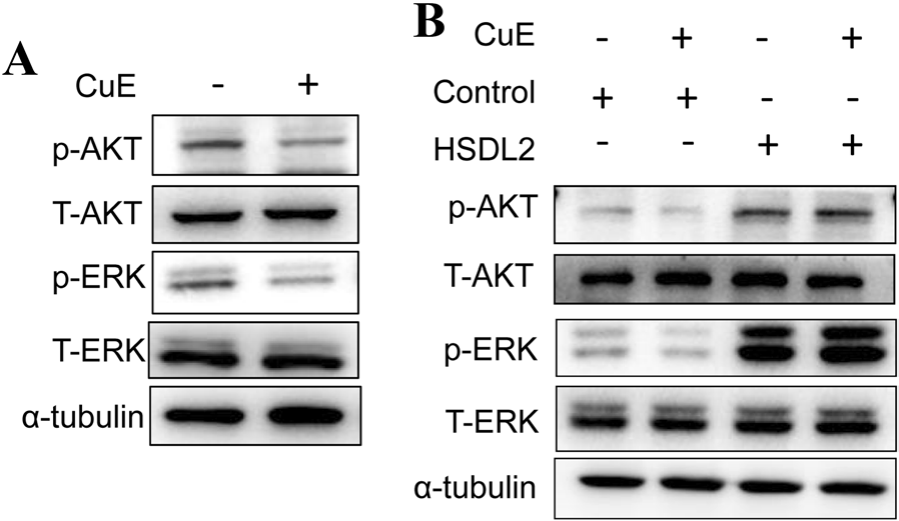
CuE inhibits the ERK and AKT signalling pathways by downregulating HSDL2. A375 cells were treated with CuE for 24 h, and the phosphorylation levels of ERK and AKT were determined by western blotting (**A**). A375 cells with or without HSDL2 overexpression were treated with CuE, and the phosphorylation levels of ERK and AKT were determined by western blotting (**B**). Data represent one of three independent experiments

Our previous data indicated that CuE could inhibit the expression of HSDL2; hence, we investigated whether CuE also affected the AKT and ERK signalling pathways. We performed western blotting assays to investigate the change in the phosphorylation levels of AKT and ERK. The data showed that CuE markedly reduced the phosphorylation levels of ERK1/2 and AKT relative to control cells (Fig. 8A). Moreover, upregulation of HSDL2 enhanced ERK1/2 and AKT phosphorylation induced by CuE (Fig. 8B). Thus, the above data suggested that CuE inhibits melanoma cells by blocking the HSDL2-mediated AKT and ERK pathways.

## Discussion

A previous study showed that HSDL2 expression was upregulated in gliomas and was positively correlated with tumour grade [22]. This suggests that HSDL2 is involved not only in tumorigenesis but also in tumour progression. However, little is known about the expression and function(s) of HSDL2 in melanoma. Our data show that HSDL2 is overexpressed in melanoma relative to adjacent normal tissue. In vitro and in vivo experiments revealed that HSDL2 is important for melanoma growth. Combined with HSDL2 knockdown and overexpression assays in melanoma cells, the data confirmed the positive role of HSDL2 in melanoma proliferation and apoptotic inhibition, suggesting that silencing HSDL2 may be a potential strategy for blocking melanoma progression.

To investigate the molecular basis for the effects of HSDL2 on melanoma cell proliferation and survival, we examined the activation status of multiple intracellular signalling proteins involved in cell survival and proliferation using stress, apoptosis, and RTK signalling arrays. HSDL2 knockdown decreased the phosphorylation of ERK1/2 (Thr202/Tyr204) and AKT (Ser473) relative to control cells. The ERK and Akt signalling pathways are constitutively activated in melanoma and play an important role in melanoma development and progression [23, 24]. Activated ERK1/2 can regulate the relative proteins involved in proliferation and apoptosis, such as Bad, Bcl-2, and C-myc, thereby contributing to melanoma growth [25, 26]. In addition, the Akt pathway is another significant pathway in melanomagenesis, promoting melanoma cell proliferation, metastasis and drug resistance, inhibiting apoptosis, and stimulating DNA mutation [27, 28] and is associated with poor survival [29]. Therefore, our results provide the first evidence of a causal relationship between HSDL2 expression and the ERK and Akt signalling pathways in human melanoma development.

Some natural compounds that could inhibit the expression of HSDL2 to slow melanoma progression would provide new prospects and methods for the treatment of patients with melanomas. Previous reports have also shown that CuE has remarkable potential in suppressing the growth of multiple cancer cell types [18, 30, 31]. CuE can inhibit cell proliferation, induce cell cycle arrest and enhance apoptotic cell death by modulating the STAT3, MAPK, PI3K/AKT, Wnt/beta-catenin and mTOR signalling pathways [17, 19, 32–34]. CuE can also suppress angiogenesis by inhibiting the VEGFR2-mediated JAK2 − STAT3 signalling pathway and cancer cell metastasis by inhibiting depolymerization of actin filaments [35, 36]. In addition, CuE can enhance the sensitivity of tumours such as gastric cancer and colorectal cancer to conventional chemotherapeutic drugs [17, 18]. Fortunately, our data demonstrated that CuE could inhibit HSDL2 expression and melanoma cell proliferation and induce cell apoptosis, a significant target for slowing tumour development, by suppressing the ERK and AKT pathways and reducing xenograft melanoma growth. Moreover, upregulation of HSDL2 slowed the antitumour activity of CuE, and weakened CuE-inhibited the phosphorylation of ERK and AKT. In sum, these results support the potential development of CuE as a therapeutic drug for melanoma.

## Conclusion

Our data demonstrated that HSDL2 is overexpressed in melanoma tissues and that its silencing blocks melanoma progression by inhibiting cell proliferation and enhancing apoptosis via inhibition of the ERK and AKT pathways. Therefore, HSDL2 may be a promising therapeutic target against melanoma. Moreover, CuE inhibited melanoma growth by blocking the HSDL2-mediated ERK and AKT pathways. Our results suggest that CuE may be a useful chemopreventive strategy for melanoma patients with high HSDL2 levels.

## Supplementary Information

**Additional file 1: Fig. S1.** CuE suppresses HSDL2 protein level. (A and B) A375 cells were treated with different doses of CuE, and the protein levels were examined by western blotting. (A) the results of the second repeat experiment; (B) the results of the third repeat experiment.

## Data Availability

The datasets used and/or analysed during the current study are available from the corresponding author on reasonable request.

## Abbreviations

Bcl-2: B-cell lymphoma/leukaemia-2
Bax: Bcl-2 associated X protein
CuE: Cucurbitacin E
ERK: Extracellular signal-regulated kinase
FBS: Foetal bovine serum
SDR: Short-chain dehydrogenase/reductase
HSDL2: Hydroxysteroid dehydrogenase-like 2
JAK: Janus kinase
MAPK: Mitogen-activated protein kinase
mTOR: Mammalian target of rapamycin
PBS: Phosphate-buffered saline
PI3K: Phosphoinositide 3-kinase
RT-qPCR: Real-time reverse transcription-quantitative polymerase chain reaction:
STAT3: Signal transducer and activator of transcription 3
VEGFR2: Vascular endothelial growth factor receptor 2
