# Supplementary figures and images for: Cucurbitacin E inhibits cellular proliferation and induces apoptosis in melanoma by suppressing HSDL2 expression
Source: Chin Med. 2022 Feb 22;17:28. doi: 10.1186/s13020-022-00582-y (PMC8862504; doi:10.1186/s13020-022-00582-y)

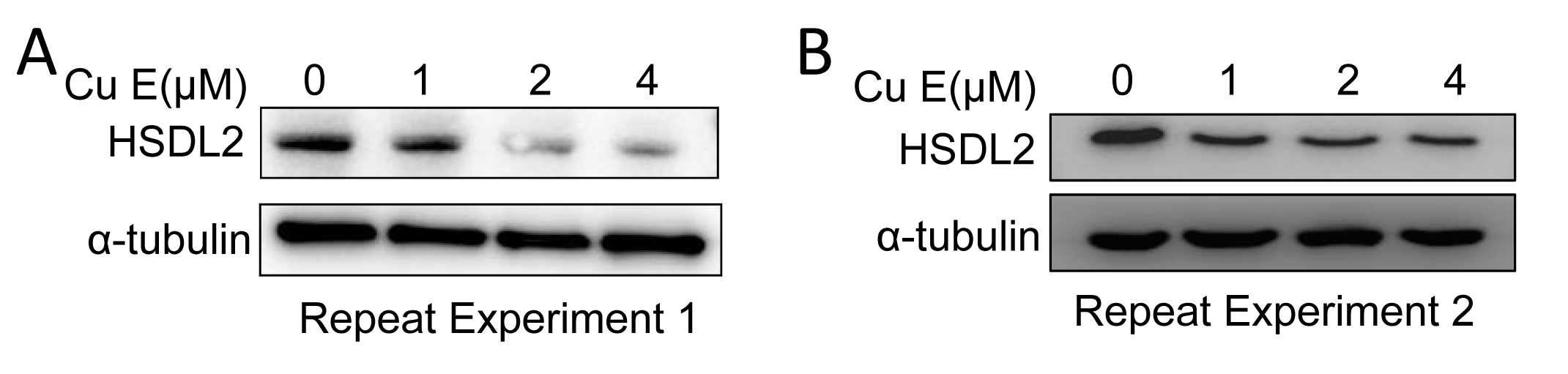

Supplement: Supplementary file 1 — Additional file 1: Fig. S1. CuE suppresses HSDL2 protein level. (A and B) A375 cells were treated with different doses of CuE, and the protein levels were examined by western blotting. (A) the results of the second repeat experiment; (B) the results of the third repeat experiment. [file 13020_2022_582_MOESM1_ESM.tif]
